# Multiscale experimental study of H$_2$/brine multiphase flow in porous rock characterizing relative permeability hysteresis, hydrogen dissolution, and Ostwald ripening

**DOI:** 10.1038/s41598-024-81720-4

**Published:** 2024-12-04

**Authors:** Maartje Boon, Tim Rademaker, Chandra Widyananda Winardhi, Hadi Hajibeygi

**Affiliations:** 1 Institute of Applied Mechanics, University of Stuttgart, 70569 Stuttgart, Germany; 2 Faculty of Civil Engineering and Geosciences, Delft University of Technology, 2600 GA Delft, The Netherlands; 3 Department of Geology, Ghent University, 9000 Ghent, Belgium

**Keywords:** UHS, Core-flood test, Hydrogen dissolution, X-ray CT, Relative permeability, Ostwald ripening, Hydrology, Energy storage

## Abstract

To safely and efficiently utilize porous reservoirs for underground hydrogen storage (UHS), it is essential to characterize hydrogen transport properties at multiple scales. In this study, hydrogen/brine multiphase flow at 50 bar and 25 °C in a 17 cm Berea sandstone rock core was characterized and visualized at the pore and core scales using micro X-ray CT. The experiment included a single drainage and imbibition cycle during which relative permeability hysteresis was measured, and two no-flow periods to study the redistribution of hydrogen in the pore space during storage periods. An end-point relative permeability of 0.043 was found at $S_w=0.56$, and the residual gas saturation was measured to be 0.32. Despite extensive pre-equilibration, significant dissolution of hydrogen into brine occurred near the core inlet due to elevated pressures and the corresponding increase in hydrogen solubility. During drainage, many disconnected hydrogen ganglia were observed further down the core which could be explained by the exsolution of the dissolved hydrogen. During imbibition, the dissolution of hydrogen led to the formation of preferential flow paths near the inlet, and eventually removed most of the trapped hydrogen in the final stage of the experiment. The two no-flow periods were characterized by the fragmentation of medium-sized hydrogen ganglia and the growth of a few larger ganglia, providing evidence for hydrogen re-connection through the dissolution-driven process of Ostwald ripening. These results demonstrate that despite the low solubility of hydrogen in brine, hydrogen dissolution can significantly influence the observed multiphase flow and trapping behavior in the reservoir and should be considered in UHS modeling.

## Introduction

Energy storage in the form of hydrogen is emerging as a key solution to overcome the intermittency and geographical constraints of renewable energy sources. However, due to the low density of hydrogen, immense storage volumes will be needed^1^. Geological porous reservoirs are widely available and provide the volumes required to store hydrogen on a large scale^2–5^. These reservoirs have demonstrated their ability to store methane, air, and carbon dioxide effectively and safely. While there is a growing interest in research on underground hydrogen storage (UHS) in porous reservoirs, the applicability of porous reservoirs for large-scale UHS still remains to be investigated. In recent years, however, several pioneering projects have started exploring this field more extensively, as evidenced by initiatives such as HyUsPRe^6^, HyChico^7^, and Underground Sun Storage^8^.

The operation of UHS is expected to be significantly different from the underground storage of other gases. This difference arises mainly due to the cyclic nature of UHS. Unlike carbon dioxide storage, hydrogen storage involves repeated cycles of injection and withdrawal, influenced by the intermittent nature of green energy production. In addition, the unique physical properties of hydrogen, such as its low density and viscosity^9^, can lead to different flow behavior in the reservoir due to a complex interaction of gravitational, capillary, and viscous forces^10^. Microbial activity^11–13^ and geochemical reactions^14^ can further impact the flow behavior, recoverability and purity of the stored hydrogen.

UHS in porous reservoirs can be divided into aquifer storage, depleted gas field storage, and depleted oil field storage^2^. In all three cases, in-situ brine is commonly present, albeit in varying amounts. A good understanding of the multiphase flow dynamics of hydrogen/brine in porous rock is therefore important for investigating the feasibility of UHS. The interaction of hydrogen with the in-situ reservoir brine and rock can be characterized by multiphase flow parameters such as relative permeability and capillary pressure^15–17^. These are important input parameters for reservoir simulators and can be derived through pore network modeling^4^ using experimentally measured contact angles to correctly capture the wettability of the hydrogen/brine/rock system^10,18–23^ or they can be directly measured during core-flood tests in the laboratory^10,22,24–26^.

The study by Yekta et al.^22^, was the first to experimentally characterize the multiphase flow behavior of the hydrogen/brine/rock system by measuring drainage capillary pressure and relative permeability in a sandstone rock. Since then, the number of experimental studies involving hydrogen/brine injection in natural rock has increased and the work has expanded to include the measurement of relative permeability hysteresis^10,24,25^ and the visualization and characterization of hydrogen/brine multiphase flow at the pore-scale^27,28^. Higgs et al.^24^, provides an overview of the different studies. An extension of this overview can be found in the supporting information. Different pressure, temperature, and injection conditions (e.g., flow rate, orientation) were used in the different studies to represent different storage depths and flow regimes. While direct comparisons are challenging due to the different rock types and inherent rock heterogeneity, one notable observation is the consistently low hydrogen endpoint relative permeability reported in all but one of the current studies (Supplementary Table 3-supporting information), which has been attributed to the low viscosity of hydrogen^24^. The endpoint relative permeability is an important indicator to evaluate the injectivity of hydrogen in rock. However, from an economic perspective, recoverability is equally important and can be characterized by the residual gas saturation, the amount of trapped gas at the end of imbibition. In the context of UHS, imbibition relative permeability and the corresponding residual gas saturation have been studied experimentally by Boon and Hajibeygi^10^, Lysyy et al.^25^, and Higgs et al.^24^. In addition, residual gas saturation in natural rock has been measured in several other core-^29^ and pore-scale experiments^27,28,30–32^. For these various core- and pore-scale studies, the residual gas saturation ranged from 0.07 to 0.41, indicating that a significant amount of stored hydrogen could become unrecoverable.

To accurately measure the residual gas saturation, it is important to use brine that is equilibrated with the gas to avoid dissolving the gas in the brine. Due to the very low solubility of hydrogen in water (0.14g/kg${_w}$ at 100 bar and 50 °C^33^), hydrogen dissolution was initially thought to be negligible. However, several studies have shown that hydrogen dissolution actually affects the observed pore-scale behavior^27,34^, which can lead to very different flow behavior at the core-scale^10^. In the core-scale study of Boon and Hajibeygi^10^, hydrogen dissolution in water combined with gravity segregation resulted in preferential channel flow for the water phase along the lower part of the core, and a significant amount of hydrogen got trapped as a result. Interestingly, gas dissolution is still observed even when the brine and gas are extensively pre-equilibrated before being injected into the porous media, as shown in the experimental investigation of Gao et al.^35^. This behavior can likely be explained by ripening dynamics. Due to capillary pressure, the pre-equilibrated brine is actually still undersaturated compared to the pore pressure of the gas. Moreover, as the gas bubble dissolves, the capillary pressure increases, leading to a greater rate of dissolution, and eventually all of the gas is dissolved and removed from the system by advection^24,35^. This makes it difficult to accurately measure residual gas saturation. Therefore, a common practice in these types of core flood tests is to determine residual gas saturation after injecting 5 pore volumes of 100% brine.

During periods of no flow, dissolved hydrogen can be transported by diffusion. Local variations in capillary pressure, and corresponding hydrogen solubility, lead to local gradients in dissolved hydrogen concentration. These gradients result in a diffusive mass flux of dissolved hydrogen from regions of high capillary pressure, i.e smaller gas bubbles, to regions of low capillary pressure, i.e. bigger gas bubbles, where the dissolved hydrogen exsolves. As a result, larger bubbles tend to grow at the expense of smaller bubbles. This process is called Ostwald ripening. Blunt^36^, investigated the time scales for reaching capillary equilibrium in porous rock through the process of Ostwald ripening and the implications on long-term gas storage and laboratory experiments. It was estimated that it would take 17, 35, 104, and 162 days, respectively, for carbon dioxide, hydrogen, methane, and nitrogen to reach equilibrium at the laboratory scale. Ostwald ripening in porous rock has been studied experimentally in recent years for the CO$_2$/brine system in the context of geological carbon dioxide storage^37^. For the hydrogen/brine/rock system, a few experimental studies exist. In Zhang et al.^32^, and Goodarzi et al.^28^, high-resolution CT imaging was used to visualize the effect of Ostwald ripening on hydrogen distribution in Bentheimer sandstone rock. Zhang et al.^32^, observed the rearrangement of trapped hydrogen ganglia after a 12 h no-flow period, the larger ganglia grew in size and the smaller ganglia tended to disappear. The same experiment was performed with nitrogen but no significant ganglia rearrangement was observed due to the different Ostwald ripening time scales for each gas. Goodarzi et al.^28^, concluded that after a 16 h period without flow, the hydrogen gathered into larger ganglia, with one large connected ganglion comprising nearly all of the volume. Reconnection of the disconnected hydrogen ganglia during storage periods is favorable for UHS because it can reduce the amount of residually trapped gas in the subsequent production period and improve hydrogen recoverability, as shown in the pore network modeling study of Adebimpe et al.^38^.

The above studies demonstrate that the hydrogen/brine system in porous rock exhibits complicated flow and transport properties due to a complex interplay of gravitational, capillary, and viscous forces combined with hydrogen dissolution in brine. Previous core-flooding experiments have been limited to either characterizing pore-scale behavior on small core plugs using high-resolution micro-CT^24,27,31^, or characterizing core-scale behavior on larger samples using low-resolution medical CT^10,25^. In this study both pore- and core-scale imaging are combined, during the same experiment, to obtain a multi-scale characterization of the behavior of the hydrogen/brine/rock system including relative permeability hysteresis, hydrogen dissolution, and Ostwald ripening.

## Material and methods

In this multi-scale study, single cycle relative permeability hysteresis and Ostwald ripening are characterized for the H$_2$/brine system by carrying out core-flood tests on a vertically oriented 17 cm long Berea sandstone rock core. The sample is imaged by utilizing an XRE CoreTOM (Tescan) XCT to visualize the distribution of hydrogen and brine in the rock at both the pore- and core-scale. The experimental apparatus and procedure of Boon and Hajibeygi^10^ are adjusted to ensure the equilibration of hydrogen and brine before the start of the experiment.

### Rock and fluids

An untreated homogeneous Berea sandstone rock core, 3 wt% KI brine, and hydrogen gas are used in the experiment. The rock core is 17 cm long and has a diameter of 10.5 mm. The core is inserted into a carbon core holder and a combination of epoxy resin (L20) and hardener (EPH 161) is injected to securely bond the core within the core holder. From CT imaging, it is observed that sealing resin slightly penetrated the surface of the core. Therefore, for the (relative) permeability calculations, a radius of 5.0 mm is used instead of 5.25 mm. The permeability and porosity of the sample, measured at the start of the experiment, are 104 mD (1.03 $\times$ 10$^{-13}$ m$^2$) and 18.3%, respectively. The pore size distribution, measured using high-resolution CT for a 1 cm long section in the center of the core, can be found in the supporting information (Supplementary Fig. 1). The hydrogen gas is produced by Linde-gas Company and has a purity of 99.99 mol%. Degassed 3 wt% KI brine is used to enhance the contrast between the three phases in the CT images. The viscosity of the brine was measured to be 9.22 $\times$ 10$^{-4}$ Pa$\cdot$s at 25 °C and atmospheric pressure. At these same conditions, the density was measured resulting in $1019.7 \hbox {kg/m}^3$ using a densimeter and $1017.4 \hbox {kg/m}^3$ using the scale density. The mean is used for calculations. The experiment is conducted at a pressure of 50 bar and 25 °C. The density of hydrogen at these conditions is $3.949 \hbox {kg/m}^3$^39^, while the viscosity of hydrogen is 8.94 $\times$ 10$^{-6}$ Pa$\cdot$s^39^.

The pore-scale capillary number (*Ca*), used to describe the balance of viscous forces over capillary forces, is given by

1 $$\begin{aligned} Ca_i=\frac{\nu _{i} \mu _{i}}{\sigma }, \end{aligned}$$

where *i* refers to the displacing phase (hydrogen or brine), $v_{i}$ is the superficial (Darcy) velocity of the displacing phase [m/s] and $\mu _{i}$ is the viscosity of the displacing phase [Pa$\cdot$s]. $\sigma$ represents the interfacial tension between the fluids [N/m], which in this experiment is presumed to be equivalent to that of hydrogen and pure deionized water under the experimental conditions (72.6 mN/m^40^). The flow rate used in the experiment is 0.5 ml/min during both drainage and imbibition, resulting in a *Ca* of 1.31 $\times$ 10$^{-8}$ for drainage and a 1.35 $\times$ 10$^{-6}$ for imbibition, which both represent capillary dominated conditions^28^. The flow rate is chosen such that the capillary numbers are similar to previous studies, facilitating meaningful comparisons.

### Experimental apparatus

**Fig. 1:**
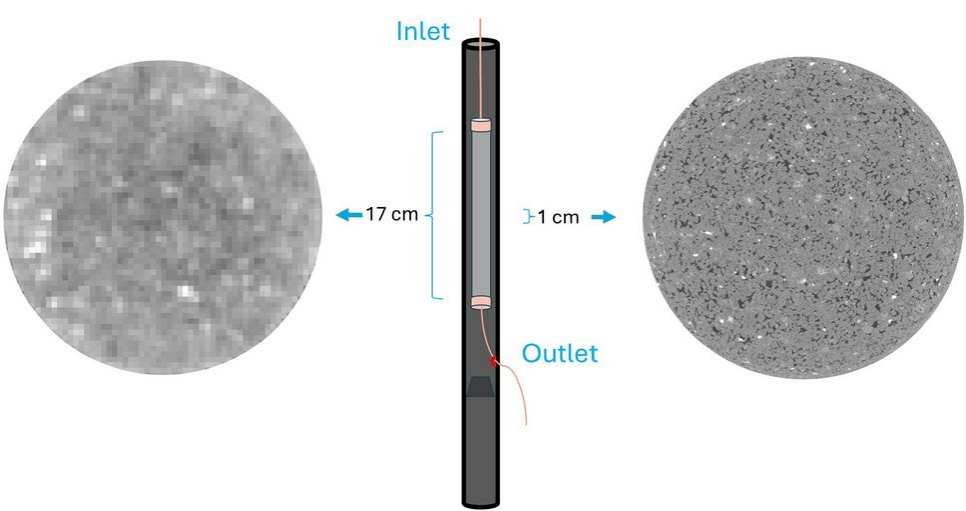
Schematic of the 17 cm Berea sample in its carbon core holder. The whole core is scanned at low resolution (175 μm). A 1 cm section in the middle of the core is scanned at high resolution (6.5 μm) to study pore-scale behavior. The circles represent a raw 2D image at the respective resolution.

The experimental apparatus is based on the apparatus used by Boon and Hajibeygi^10^ with the primary differences being the use of the CoreTOM micro-CT scanner, the creation of a closed-loop system for both fluids, and addition of a by-pass loop to carefully pre-equilibrate the hydrogen and brine before the start of the experiment. The CoreTOM micro-CT scanner, manufactured by TESCAN, is capable of imaging large samples at high resolution. In this experiment, the full length of the core is imaged at low resolution (macro-scale) to visualize the saturation distribution, while a 1 cm long section at the middle of the core is imaged at high resolution (micro-scale) to observe pore-scale behavior (Fig. 1). The scanning parameters of both macro- and micro-scale scans are summarized in Table 1.

**Table 1:** CT scanning parameters.

Setting	Macro-scale scans	Micro-scale scans
Exposure time [s]	34	175
No. of projections	1440	2880
Set voltage [kV]	70.00	70.00
Actual power (range) [kW]	50.01–50.11	15.01–15.04
Rotation angle [$^\circ$]	360	360
Filter	1.5 mm Al	1.5 mm Al
Voxel size [µm]	175.0	6.5
Scanned length [mm]	1925	13

To ensure the integrity of the experiment, a by-pass loop is used to carefully equilibrate the hydrogen and the brine before the start of the experiment, which, together with the closed-loop system for both fluids, minimizes the risk of introducing non-equilibrated brine or hydrogen. Two pulse-free high-precision piston pumps, manufactured by Vindum Engineering, Inc. are used to inject the equilibrated brine and hydrogen. These pumps exhibit an accuracy of 0.1% and are connected to a brine container and a hydrogen cylinder for the initial filling of the pumps. A 3 m long PEEK tubing (0.75 mm inner diameter) serves as the conduit connecting the apparatus outside the CT scanner to the inlet pressure transducer (UNIK 5000), which is inside the CT scanner. A 1.25 m Radel tubing (0.75 mm ID) connects the pressure transducer to the inlet of the core. The core is placed vertically inside the CT scanner and the fluids are injected at the top of the core. A 0.78 m Radel tubing (0.75 mm ID) is used to connect the outlet at the bottom of the core to the second pressure transducer. A 3 m PEEK tubing (0.75 mm ID) connects outlet pressure transducer to an additional Vindum pump positioned outside the CT scanner. This supplementary pump plays a crucial role in upholding a consistent back pressure within the system. Positioned between the core outlet and the separation vessel, it acts as a buffer to reduce pressure fluctuations caused by the pump-filling process. The separation vessel has a volume of 150 ml and serves to segregate the brine and hydrogen, which subsequently replenish the pumps, facilitating the formation of a closed-loop system. The system is brought to an operational pressure of 50 bar using a nitrogen cylinder and a back-pressure regulator. Upon completion of the experiment, an effluent bottle connected to the back-pressure regulator collects the brine. Subsequently, the effluent bottle is replaced with a tube connection leading to a fume hood for the safe disposal of hydrogen.

**Fig. 2:**
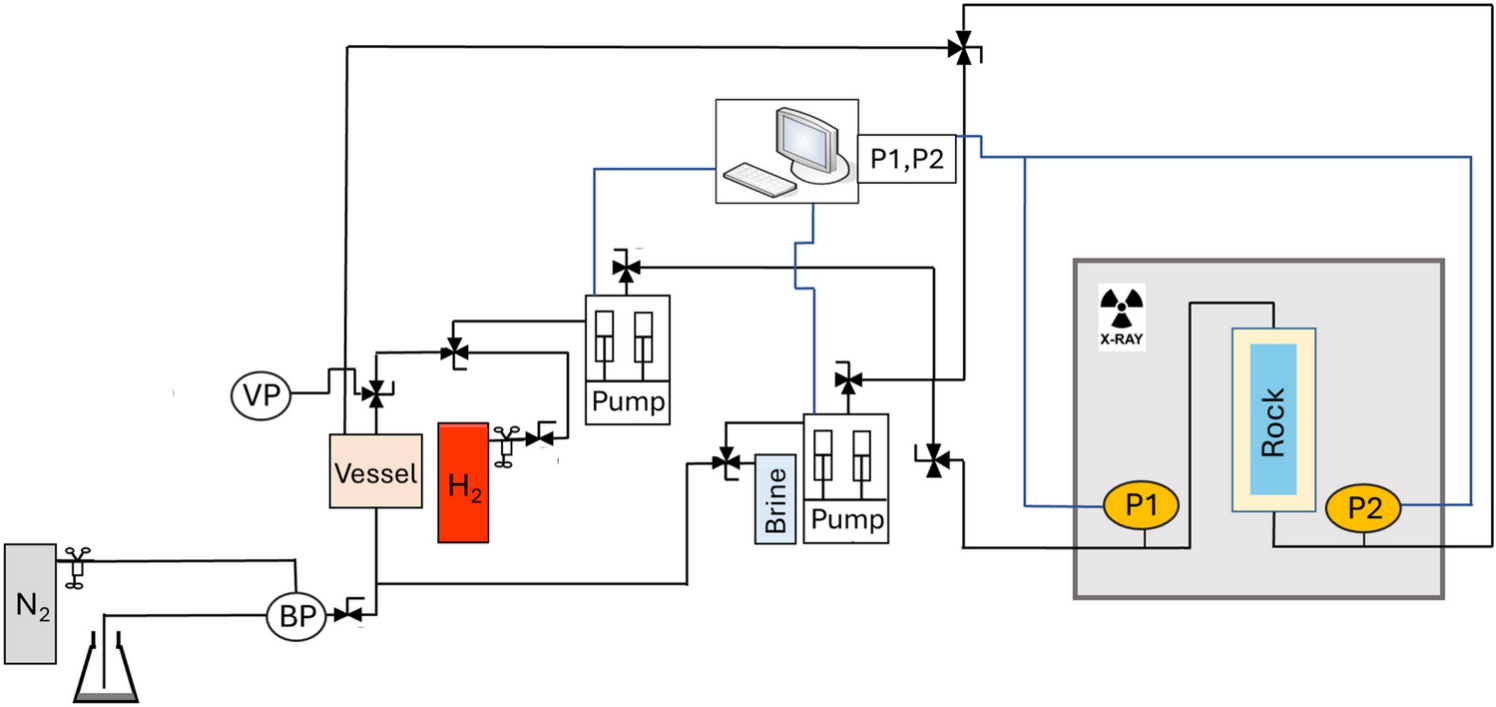
Schematic of the experimental apparatus. Here, *BP* is back pressure, *VP* is vacuum pump, and *P1,P2* are pressure transducer 1 and 2, respectively. Notice that the rock sample is placed vertically, to prototype the real-field injection scenario from the top of the reservoir.

Safety measures are paramount throughout the experiment. A hydrogen monitor is installed within the CT scanner, and the hydrogen cylinder remains closed except during the filling of the hydrogen pump. The maximum system volume of hydrogen is 180 ml when the system is completely saturated. Throughout the remainder of the experiment, the volume of hydrogen gas remains below 100 ml. The experiment is conducted in a well-ventilated area. A schematic of the experimental apparatus can be found in Fig. 2.

### Experimental procedure and analysis

The experiment involved a single drainage and imbibition cycle to measure relative permeability hysteresis. Two no-flow periods were incorporated into the experiment to characterize the redistribution of hydrogen gas as the result of Ostwald ripening. A detailed description of the experimental procedure and analysis is given below.

#### Background scans, absolute permeability test, and pre-equilibration

At the start of the experiment, background scans are taken at both the micro- and macro-scale (Table 1). The macro-scale images are used to determine porosity and saturation, while the micro-scale images are used for the pore-scale image analysis. In this process, the first step is scanning the dry core (air saturated) at both the micro- and macro-scale. Next, air is removed from the system with a vacuum pump after which the core is saturated and pressurized with hydrogen up to 50 bar while scans are taken at both micro- and macro-scale. This is followed by gradually reducing the back-pressure to atmospheric while venting hydrogen into the fume hood. Subsequently, the core is flushed with CO_2_, vacuumed, and saturated with brine while increasing the system pressure to 50 bar before taking scans at both micro- and macro-scale.

The porosity of the core is obtained from the air-saturated and brine-saturated scans using

2 $$\begin{aligned} \phi =\frac{C T_{{brine}}-C T_{d r y}}{I_{{brine}}-I_{{air}}}. \end{aligned}$$

Here, the normalized CT values for the brine saturated and dry core are indicated by $CT_{brine}$ and $C T_{d r y}$, respectively. The normalized CT values that would have been obtained if air and brine alone had been scanned are $I_{brine}$ and $I_{air}$^16^, and are here obtained by averaging the CT values of 27 brine-filled voxels and 27 air-filled voxels in the brine-saturated core and air-saturated core, respectively. In the case of medical X-ray CT, CT values are standardized using dimensionless Hounsfield units [HU], where air and water correspond to -1000 HU and 0 HU, respectively. In the CoreTom CT scanner, the CT values are not standardized. Therefore, to calculate porosity and saturation from the macro-scale background scans all scans were normalized with respect to the equilibrated-brine background scan, using the end-caps of the core.

The next step in the experimental procedure is the absolute permeability measurement for which brine is injected for a range of flow rates (between 0.5 and 2 ml/min) while measuring the pressure drop along the core. The absolute permeability can then be determined with the use of Darcy’s law,

3 $$\begin{aligned} q=\frac{A k}{\mu } \left( \frac{\Delta P}{L}+ \rho g\right) , \end{aligned}$$

here *q* is the injected flow rate [$\hbox {m}^3$/s], *A* is the cross-sectional area of the core [$\hbox {m}^2$], *k* is the absolute permeability [$\hbox {m}^2$], $\mu$ is the viscosity of the fluid [Pa$\cdot$s] and $\Delta P$ is the pressure drop [Pa] over length *L* [m]. To take into account the vertical orientation of the core with the inlet at the top, the impact of gravity is included, where $\rho$ is the fluid density [$\hbox {kg/m}^3$], and *g* is the gravitational acceleration^22^.

To avoid hydrogen dissolution into brine, the hydrogen and brine must be pre-equilibrated at the experimental conditions before the start of the multi-phase flow experiment. To ensure this, both fluids are pumped through the system while bypassing the core at a rate of 10 ml/min each for 20 min at 50 bar. The circulated volume (400 ml) exceeds twice the system volume of 180 ml. Next, the hydrogen pump is stopped and the equilibrated brine is recirculated over the core, again flowing through the partly hydrogen filled separation vessel to further ensure the equilibration of hydrogen and brine. As final step before the multi-phase flow experiment, a scan is made of the equilibrated-brine-saturated core at both the micro- and macro-scale.

#### Relative permeability measurements

The equilibrated-brine-saturated core serves as the initial stage for the drainage relative permeability experiment for which the steady-state method is employed^16^. This involved the simultaneous injection of the wetting and non-wetting fluids into the core at varying fractional flows. A consistent flow rate of 0.5ml/min is maintained throughout the experiment. The inlet and outlet pressures are continuously monitored and recorded throughout the process. The attainment of steady-state is indicated by a constant pressure drop ($\Delta P$) along the core. At each steady-state condition during the experiment, a macro-scale scan is conducted to determine the phase saturation according to^10^

4 $$\begin{aligned} S_{H_2}=\frac{C T_{exp }-C T_{br,eq}}{C T_{H_2}-C T_{br,eq}}. \end{aligned}$$

Here, $CT_{exp}$ represents the normalized CT values obtained from the core at a specific step in the experiment. Similarly, $CT_{H_2}$ and $CT_{br,eq}$ are the normalized CT values for the hydrogen-saturated core and the equilibrated-brine-saturated core (background scans), respectively. The water saturation is obtained with $S_w = 1- S_{H_2}.$ During drainage, the fractional flow of hydrogen ($f_{H2}$) is incrementally increased. The last increment, when only hydrogen is injected into the core ($f_{H2}$ = 1), marks the end of the drainage experiment, establishing the irreducible brine saturation ($S_{wi}$). This step also initiates the imbibition experiment, in which $f_{H2}$ is incrementally reduced. The methodology mirrors that of the drainage experiment and the end-point ($f_{H2}$ = 0) yields the residual gas saturation $S_{gr}$. The core average saturations and pressure differentials along the core observed at each steady-state condition are utilized to derive the drainage and imbibition relative permeability curves for brine and hydrogen according to Darcy’s law, extended for a multiphase system, i.e.,

5 $$\begin{aligned} q_i=\frac{A k k_{r, i}\left( S_i\right) }{\mu _i} \left( \frac{\Delta P}{L} + \rho _i g\right) , \end{aligned}$$

where *i* refers to the phase (hydrogen or brine), $k_r,_i(S_i)$ is the relative permeability of phase *i* as a function of saturation [−]. Note that there is a pressure drop over the inlet and outlet tubing between the pressure transducers and the core (See Fig. 2). However, during the drainage and imbibition experiment, the influence of the tubing on $\Delta P$ is less than 0.02 bar following the Hagen-Pousseuille equation. This is negligible to the measured $\Delta P$ during the experiment. Therefore, it is not included in the relative permeability calculations. The experimental conditions during each step of the relative permeability experiment can be found in Table 2.

The drainage relative permeability curves can be fitted to empirical functions for both the brine (Eq. 6) and hydrogen (Eq. 7). In this study, the results are fitted using the modified Brooks-Corey equations^24^, i.e.,

6 $$\begin{aligned} k_{r,w}= & k_{r,w, max }\left[ \frac{{S}_w-{S}_{w i}}{1-{S}_{g r}-{S}_{w i}}\right] ^m, \end{aligned}$$

7 $$\begin{aligned} k_{r,g}= & k_{r,g, max }\left[ \frac{\left( 1-{S}_w\right) -{S}_{g r}}{1-{S}_{g r}-{S}_{w i}}\right] ^n, \end{aligned}$$

where $k_{r,w, max }$ and $k_{r,g, max }$ are the end-point relative permeability for brine and hydrogen respectively, ${S}_{w i}$ is the irreducible brine saturation, ${S}_{g r}$ is the residual gas saturation, *m* is the water/brine relative permeability exponent and *n* is the gas relative permeability exponent.

**Table 2:** Total injection rate ($q_T$), hydrogen fractional flow ($f_{H2}$), water fractional flow ($f_{w}$), pressure drop at steady-state (*dP*), brine saturation ($S_w$), relative permeability values for hydrogen ($k_{r,H2}$) and water ($k_{r,w}$), and the number of pore volumes (PV) injected until steady-state, for each step in the experiment.

	$q_T$ [ml/min]	$f_{H2}$	$f_{w}$	*dP* [bar]	*dP* [Pa]	$S_w$	$k_{r,H2}$	$k_{r,w}$	PV
Drainage									
1	0.5	0.1	0.9	13.36	1.34E+06	0.72	1.17E−04	1.08E−01	31
2	0.5	0.3	0.7	12.07	1.21E+06	0.65	3.87E−04	9.30E−02	11
3	0.5	0.5	0.5	10.35	1.04E+06	0.65	7.52E−04	7.74E−02	5
4	0.5	0.7	0.3	7.04	7.04E+05	0.63	1.55E−03	6.83E−02	4
5	0.5	0.9	0.1	2.76	2.76E+05	0.63	5.08E−03	5.78E−02	4
6	0.5	0.97	0.03	1.04	1.04E+05	0.59	1.45E−02	4.56E−02	4
7	0.5	1	0.0	0.36	3.60E+04	0.56	4.32E−02	0	4
Imbibition									
8	0.5	0.9	0.1	2.39	2.39E+05	0.60	5.86E−03	6.77E−02	9
9	0.5	0.5	0.5	9.73	9.73E+05	0.63	8.00E−04	8.26E−02	6
10	0.5	0.1	0.9	14.54	1.45E+06	0.67	1.07E−04	9.95E−02	4
11	0.5	0.03	0.97	15.88	1.59E+06	0.68	2.94E−05	9.82E−02	4
12	0.5	0	1	15.35	1.54E+06	0.68	0	1.05E−01	3

#### Pore-scale observations

Two no-flow periods are incorporated in the relative permeability experiment, the first one after the first drainage fractional flow ($f_{H2}$ = 0.1) and the second one at the end of imbibition ($f_{H2}$ = 0.0), mimicking periods where hydrogen is stored in the reservoir. To investigate pore-scale behavior during these no-flow periods, in particular the process of Ostwald ripening, high resolution scans are taken for a 1 cm long section in the center of the core. At the end of $f_{H2}$ = 0.1 the flow is paused for 39 h and micro-scale images are captured right after pausing the flow, and right before resuming the drainage experiment. At the end of $f_{H2}$ = 0.0 the flow is paused for 92 h and micro-scale images are obtained right after pausing the flow, after 17 h, and after 92 h without flow. Please note that the duration of the storage periods partly depended on the availability of technicians authorized to perform the X-ray CT scans. To estimate the progression of Ostwald ripening, the timescale for pore-scale equilibrium in laboratory experiments is estimated using the equation presented by Blunt^36^, i.e.,

8 $$\begin{aligned} t=\frac{l^2 r \phi S_{g r} \rho _g}{2 D H \sigma m_g}, \end{aligned}$$

where *l* is the length over which equilibrium is reached [m], *r* is the typical throat radius [m], $\phi$ is the porosity of the rock, $S_{g r}$ is the residual gas saturation, $\rho _g$ is the density of the gas [$\hbox {kg/m}^3$], *D* is the diffusion coefficient [$\hbox {m}^2$/s], $\sigma$ represents the interfacial tension between the fluids [N/m], and $m_g$ is the molecular mass of the gas [kg/mol].

#### Pore-scale image analysis

**Fig. 3:**
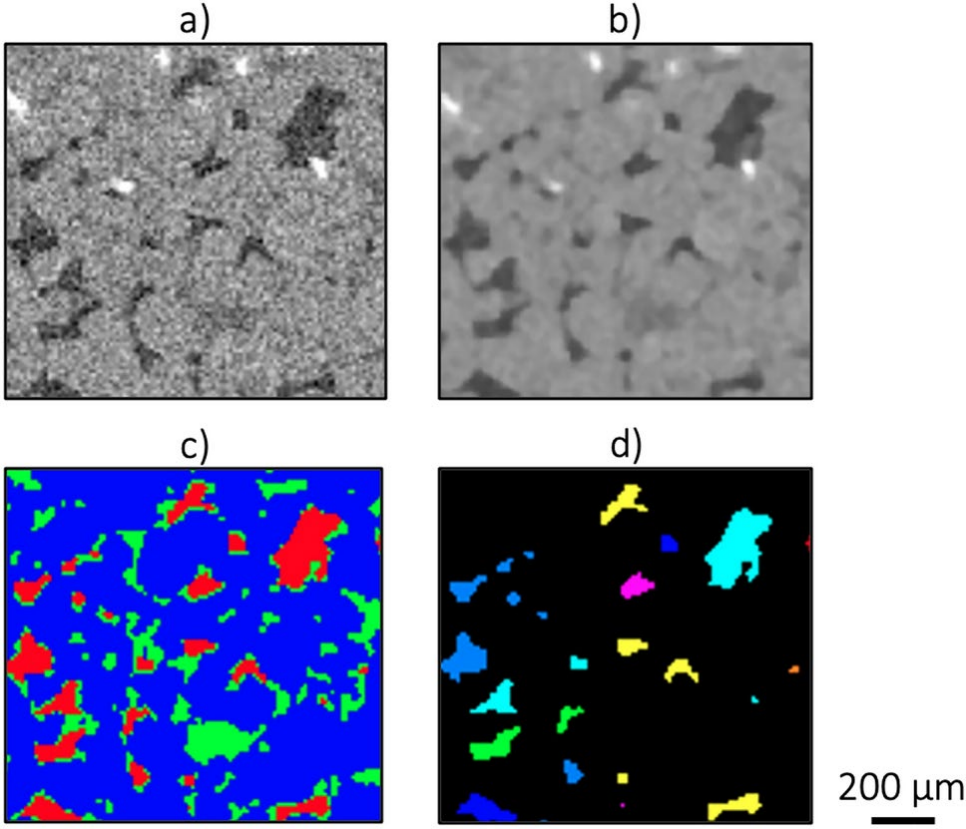
A 2D representation of the image processing procedure. (**a**) Shows a section of a raw image with hydrogen (dark grey), brine (medium grey), grains (light grey) and high density minerals (white). (**b**) Shows a section of a filtered and resampled image. (**c**) Shows a section of a masked and segmented image with hydrogen (red), brine (green) and grains and minerals (blue). (**d**) Shows the labeled distinct hydrogen ganglia where a difference in color represents non-connected ganglia.

**Fig. 4:**
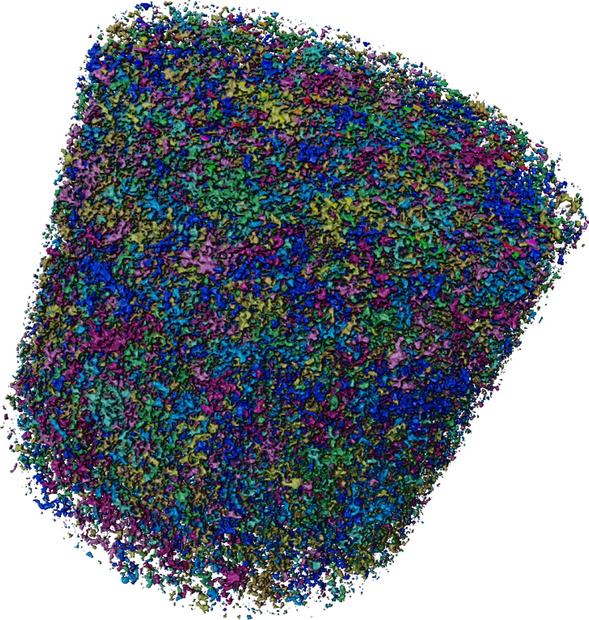
3D image showing hydrogen ganglia immediately after pausing the flow during drainage at $f_{H2}$ = 0.1. A difference in color represents non-connected ganglia. Note that the colormap consists of only 8 colors, so distant ganglia with the same color often do not represent connected ganglia.

The distribution of hydrogen ganglia volume, which changes over time, must be extracted to quantify the effect of Ostwald ripening. Several image processing steps (Fig. 3) were performed using Avizo 3D 2023 (ThermoFisher) software on all pore-scale scans. First, the core was cropped from the core holder, followed by the application of a median filter to reduce noise. To ensure proper analysis, all scans were aligned using image registration, utilizing the dry scan as the reference. The registered scans were then resampled to match the reference scan coordinate system. Once aligned, the pore space is extracted using a mask of the dry scan, leaving only the hydrogen and brine. A manual segmentation with masking grey-value of 4500 is then applied to distinguish the hydrogen from the brine within the pore space. Finally, the disconnected hydrogen ganglia are labeled, and measured individually. This allows for the determination of the volumes of discrete ganglia. The volume distributions of these ganglia serve as a measure of Ostwald ripening. To maintain reliability in the measurements, all ganglia with a size of less than 10 voxels^28^ are excluded from the ganglia volume distributions as they may be attributed to noise. An example of a reconstructed 3D image showing hydrogen ganglia is provided in Fig. 4.

## Results and discussion

### Flow characteristics and the impact of dissolution

**Fig. 5:**
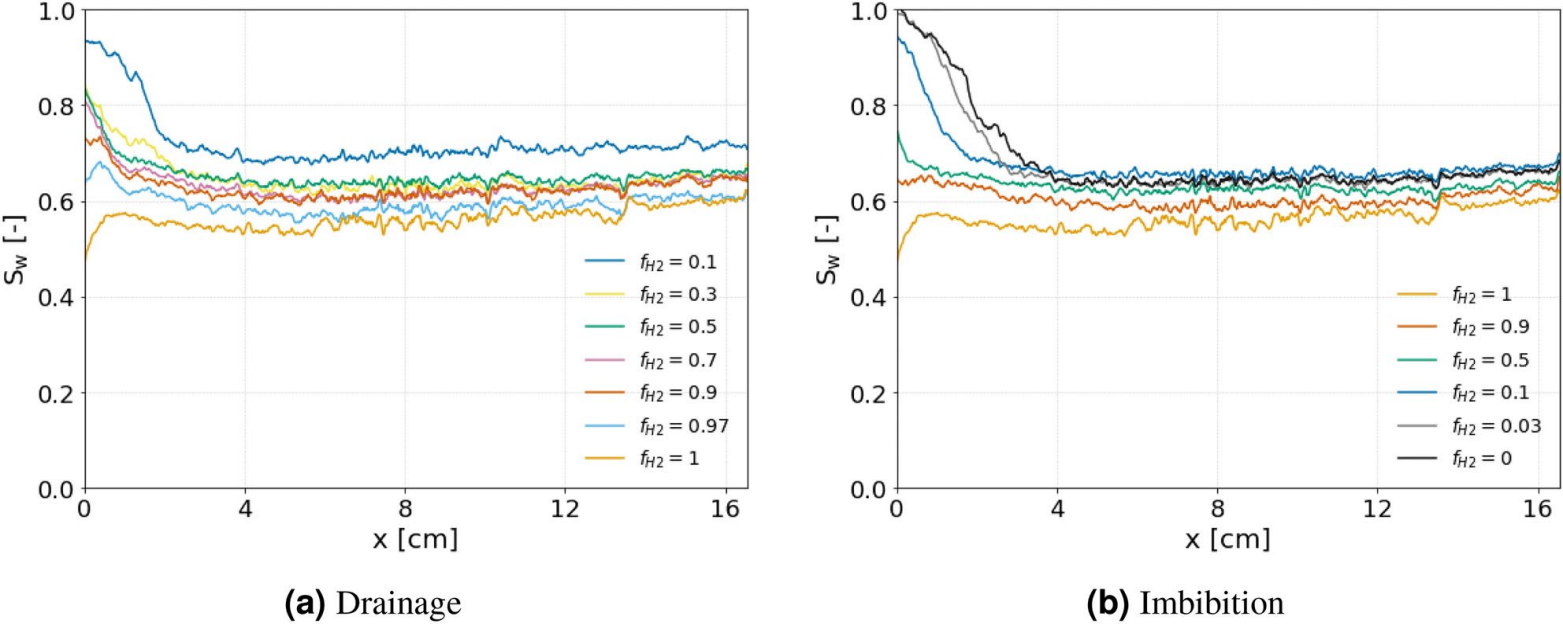
Steady-state water saturation (S$_{w}$) profiles obtained for the different fractional flows of H$_2$ ($f_{H2}$) during the relative permeability experiment for (**a**) drainage and (**b**) imbibition. The x-axis limits are set to include only the length of the core, excluding the endcaps.

**Fig. 6:**
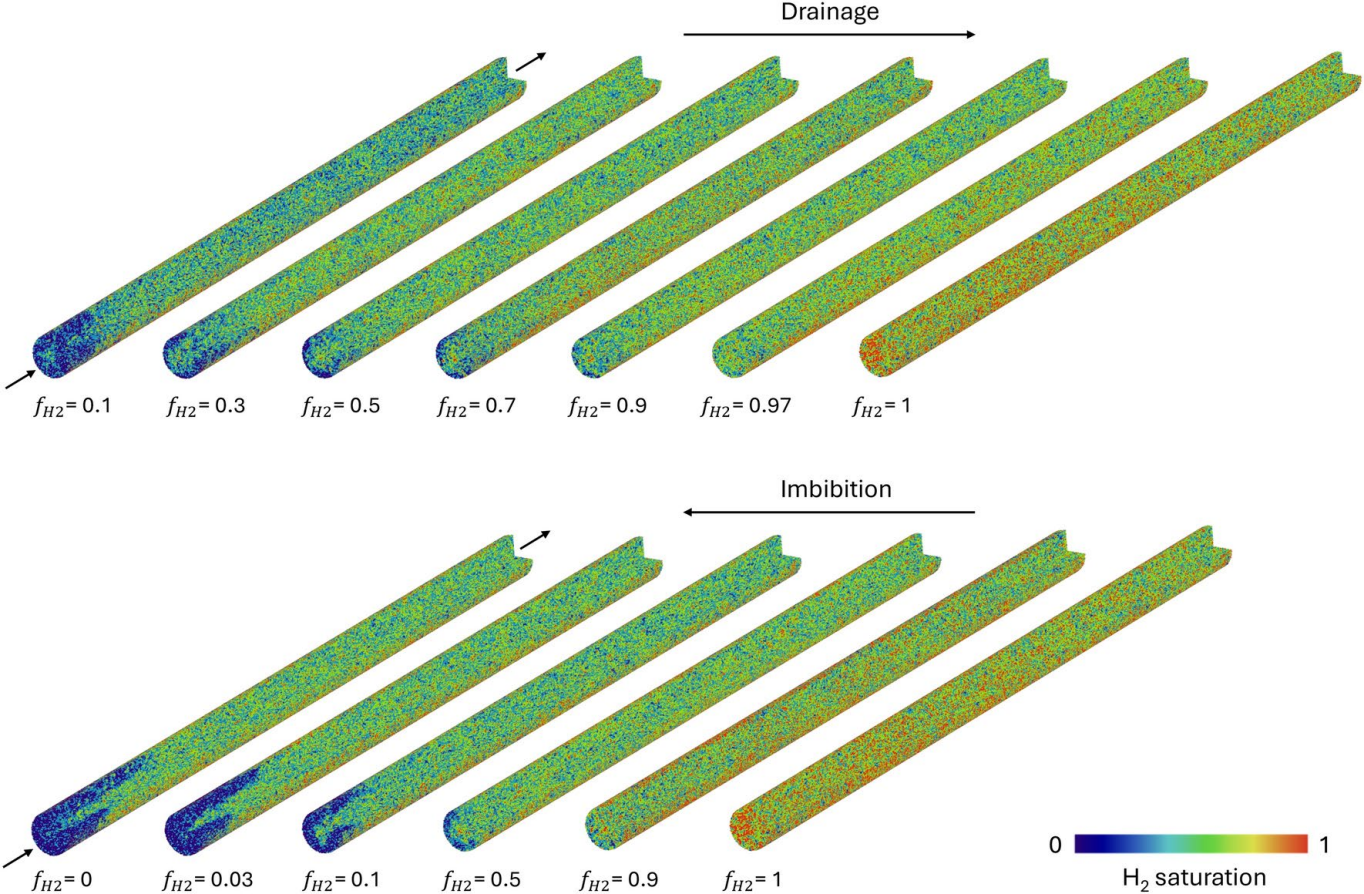
Steady-state 3D saturation maps for each step of the drainage and imbibition relative permeability experiment. Note that the core was vertically oriented during the experiment with the inlet at the top.

Figure 5 shows the hydrogen saturation profiles for each fractional flow during drainage and imbibition, while the 3D hydrogen saturation maps can be seen in Fig. 6. Interestingly, despite hydrogen’s very low solubility in brine and the extensive recirculation outside the core to pre-equilibrate the phases before the start of the experiment, the first 2 cm from the inlet exhibits a higher $S_w$ compared to the bulk of the core for many of the fractional flows, in particular the low $f_{H2}$, due to dissolution of hydrogen into the brine. Other interesting observations are the high gas saturation ($S_g=0.28$) that was reached during the first drainage step when 90% water and only 10% hydrogen was injected, and the presence of many disconnected hydrogen ganglia at the end of this fractional flow (See Fig. 4). The observed dissolution at the inlet of the core is likely due to the pressure difference between the pre-equilibration pressure of 50 bar and the actual pressure at the inlet. The pressure in the core is highest at the inlet and gradually decreases to 50 bar along the length of the core. According to Henry’s law, the higher partial pressure of hydrogen at the inlet leads to increased dissolution. As the dissolved hydrogen moves further down the core, the partial gas pressure decreases, potentially allowing hydrogen to come out of solution. This could explain the many disconnected ganglia and the resulting high gas saturation observed at the end of the first drainage step. Other explanations could be that these disconnected ganglia result from a snap-off process, or that the connections between the different ganglia are below the resolution of our images (6.5 μm). In any case, disconnected ganglia and very thin connections between the different ganglia will obstruct flow. This explains the relatively large pressure drops observed in the experiment (up to 18 bar for drainage, and 15.9 bar for imbibition) and the unexpectedly slow progression to steady-state during the first step of the drainage experiment (31 PV). It is important to mention that the total injection rate was initially set to 1 ml/min. This was reduced to 0.5 ml/min when the pressure drop reached 18 bar during the first drainage step to prevent failure of the core-holder.

The 3D saturation maps in Fig. 6 clearly show higher brine saturations near the inlet. In the bulk of the core, away from the inlet, no flow segregation is detected, indicating that both phases are flowing through the same pore space and suggesting that the extracted flow parameters are representative of a multiphase system. However, in the final stages of imbibition, preferential flow paths for the brine become apparent at the inlet away from the core’s center. Hydrogen buildup in the center of the inlet endcap forced the brine to enter from the annular regions of the inlet. Consequently, more hydrogen dissolves in the annular regions near the inlet of the core, lowering the hydrogen saturation and increasing the brine relative permeability, correspondingly. As a result, a preferential flow path forms for the brine phase. Similar behavior was observed in the core-scale study of Boon and Hajibeygi^10^. The radial segregation observed in the 2D images of Fig. 3 of the study of Goodarzi et al.^28^, suggests similar behavior occurs at the pore-scale.

The core average saturation at the end of drainage represents the minimum brine saturation in this experiment at $S_w$ = 0.56, which also corresponds to the initial gas saturation ($S_{g i}$ = 0.44). It is important to note that this saturation does not represent the actual irreducible brine saturation ($S_{w i}$) as flow rates were not adjusted to maximize brine removal from the core. The saturation at the end of imbibition ($S_{g r}$ = 0.32) represents trapped hydrogen. It is common practice to measure the residual gas saturation after injecting 5 PV of brine, here, however, the gas saturation after injecting 3 PV of brine is used as the residual gas saturation.

**Fig. 7:**
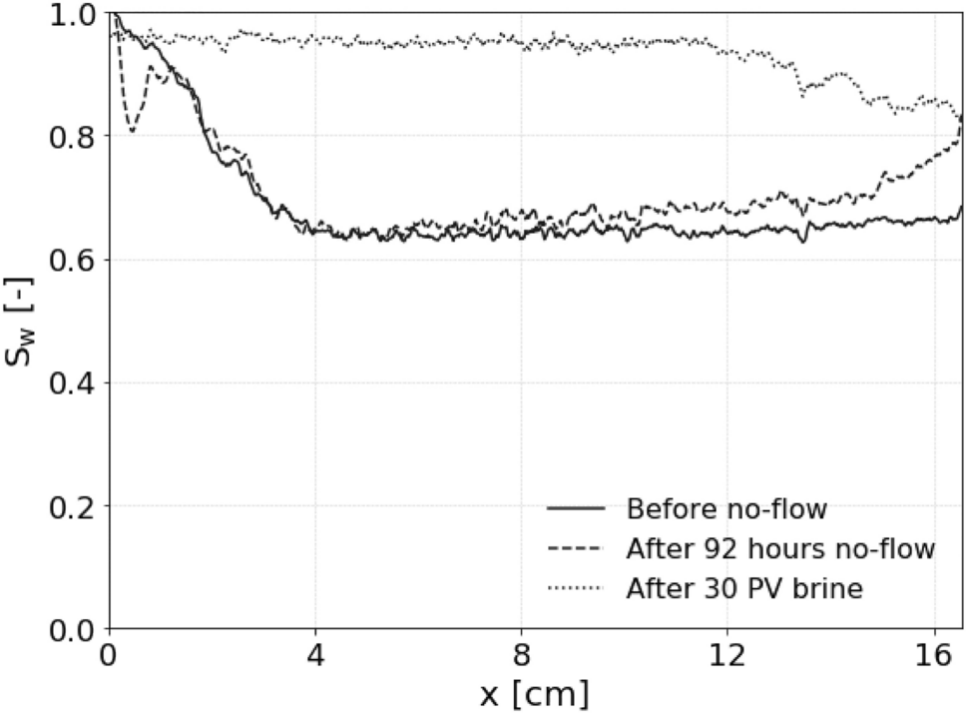
Water saturation (S$_{w}$) profiles at the start of the no-flow period following the end of the relative permeability experiment (solid line), after 92 h of storage (dashed line), and after injecting 30PV of brine (dotted line), showing almost complete removal of the residually trapped hydrogen.

In this study, the last step of imbibition involved a no-flow period of 92 h after the initial injection of 3 PV of 100% brine, followed by the injection of another 30 PV of brine until steady-state was reached. The saturation profiles measured during this last step of imbibition can be seen in Fig. 7. While there was some rearrangement of the hydrogen and possibly some diffusion towards the bottom outlet at the end of the no-flow period, the average saturation remained similar and no indication of buoyancy-driven flow was observed, as this would have led to a build-up of hydrogen at the top (inlet) of the core. During the subsequent injection of brine, a gradual decrease in the pressure drop and an increase in $S_w$ to 0.93 was observed until finally after injecting 30 PV steady-state was reached. This slow transition towards steady-state indicates that the majority of the hydrogen was removed through the process of dissolution, similar to the study of Gao et al.^35^. However, here this is more likely explained by the large pressure drops observed during the experiment instead of the higher pore-pressure of the hydrogen phase due to the capillary pressure, as suggested by Gao et al.^35^, because the capillary pressure, based on the capillary pressure measured for the Berea sandstone in Boon and Hajibeygi^10^, is at least an order of magnitude lower than the pressure drops.

After injecting 30 PV brine in this experiment, $S_{H2}$ decreased from 0.29 to 0.07, indicating that 0.22 PV of hydrogen was removed. For hydrogen in pure water at the experimental conditions (50 bar and 25$^{\circ }$C), the solubility is 6.85 $\times$ 10$^{-4}$ (salt-free mole fraction), while at 62 bar, which is the peak inlet pressure during this step of the experiment, the solubility is 8.46 $\times$ 10$^{-4}$ (salt-free mole fraction)^33^. Considering the molar mass of hydrogen gas (2.016 g/mol) and pure water (18.02 g/mol), these mole fractions correspond to a solubility of just 0.077 g/kg$_w$ and 0.095 g/kg$_w$, respectively. The solubility at the peak inlet pressure is only 0.018 g/kg$_w$ higher. Still, given hydrogen’s low density, injecting 30 PV of brine equilibrated at 50 bar (the pressure at the outlet) at elevated (inlet) pressures could dissolve a significant amount of trapped hydrogen. Based on the pressure profile of the inlet (Supplementary Fig. 2-supporting information), this value was estimated to be 0.08 PV. The additional 0.14 PV that was removed could possibly be explained by the fact that the residual gas saturation was not completely reached after the initial injection of 3 PV of brine before the start of the no-flow period, or by the dissolution-driven process of Ostwald ripening during the 92 h no-flow period, which might have reconnected and mobilized some of the trapped hydrogen. It is important to note that hydrogen has lower solubility in KI brine than in pure water due to the salting-out effect^33,41^. One of the findings by Hulikal et al.^41^ is that increasing molality of NaCl in water by 1 mol/kg$_w$, will result in a decrease of around 15% in hydrogen solubility, regardless of the pressure and temperature. Although different salts exhibit varying salting-out effects, the KI brine molality of just 0.18 mol/kg$_w$ in this experiment is not likely to have a significant impact on the observed dissolution.

#### Relative permeability

**Fig. 8:**
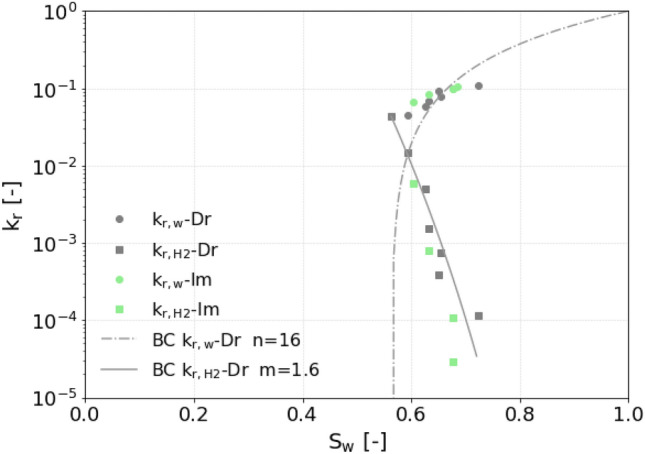
Steady-state drainage and imbibition relative permeability measurements. The modified Brooks-Corey model (Eqs. 6 and 7) is fitted to the drainage measurements using the minimum $S_w$ as $S_{wi}$.

Figure 8 presents the relative permeability curves for drainage and imbibition following the data points in Table 2. The height of the crossover point between the brine and hydrogen curves indicates the degree of interference between these two phases, leading to smaller relative permeabilities. Consistent with the findings of Yekta et al.^22^, Boon and Hajibeygi^10^, Lysyy et al.^25^, and Higgs et al.^24^, this crossover point lies at a value between 0.01 and 0.1. However, in contrast to Higgs et al.^24^, the crossover point in our data is shifted further to the right, suggesting a stronger water-wet system. This observation aligns more closely with Boon and Hajibeygi^10^, likely due to the use of the same rock type (Berea). Additionally, our study reveals similar curves for the wetting phase (brine) relative permeability during both drainage and imbibition, indicating minimal hysteresis. However, hysteresis is observed in the hydrogen relative permeability with $S_{g r}$ = 0.32. The hydrogen endpoint relative permeability found in this experiment, which is a crucial factor in evaluating injectivity in reservoir rock, is 0.043. This value is consistent with the values found in the aforementioned studies. Only Rezaei et al.^26^ found a much larger end-point relative permeability.

### Ostwald ripening

Two no-flow periods were incorporated in the experiment, the first took place after pausing the flow during drainage at $f_{H2}$= 0.1 and the second after the end of imbibition at $f_{H2}$= 0, mimicking periods of storage in the reservoir. During these no-flow periods, high-resolution scans were taken of a 1 cm section in the middle of the core to observe pore-scale behavior, in particular the process of Ostwald ripening. Table 3 lists the storage times and saturations measured during the no-flow periods for the pore-scale scans. Note that saturations may change slightly during storage due to influx or outflux of hydrogen from adjacent sections in the core.

**Table 3:** Storage time and saturations for the scans taken after pausing the flow during drainage at the end of $f_{H2}$= 0.1 (no-flow period 1), and after pausing the flow at the end of the imbibition relative permeability experiment at $f_{H2}$= 0 (no-flow period 2).

No-flow period	Scan	Storage time	S$_w$
1	1	–	0.69
	2	39 h	0.66
2	1	–	0.61
	2	17 h	0.64
	3	92 h	0.65

**Fig. 9:**
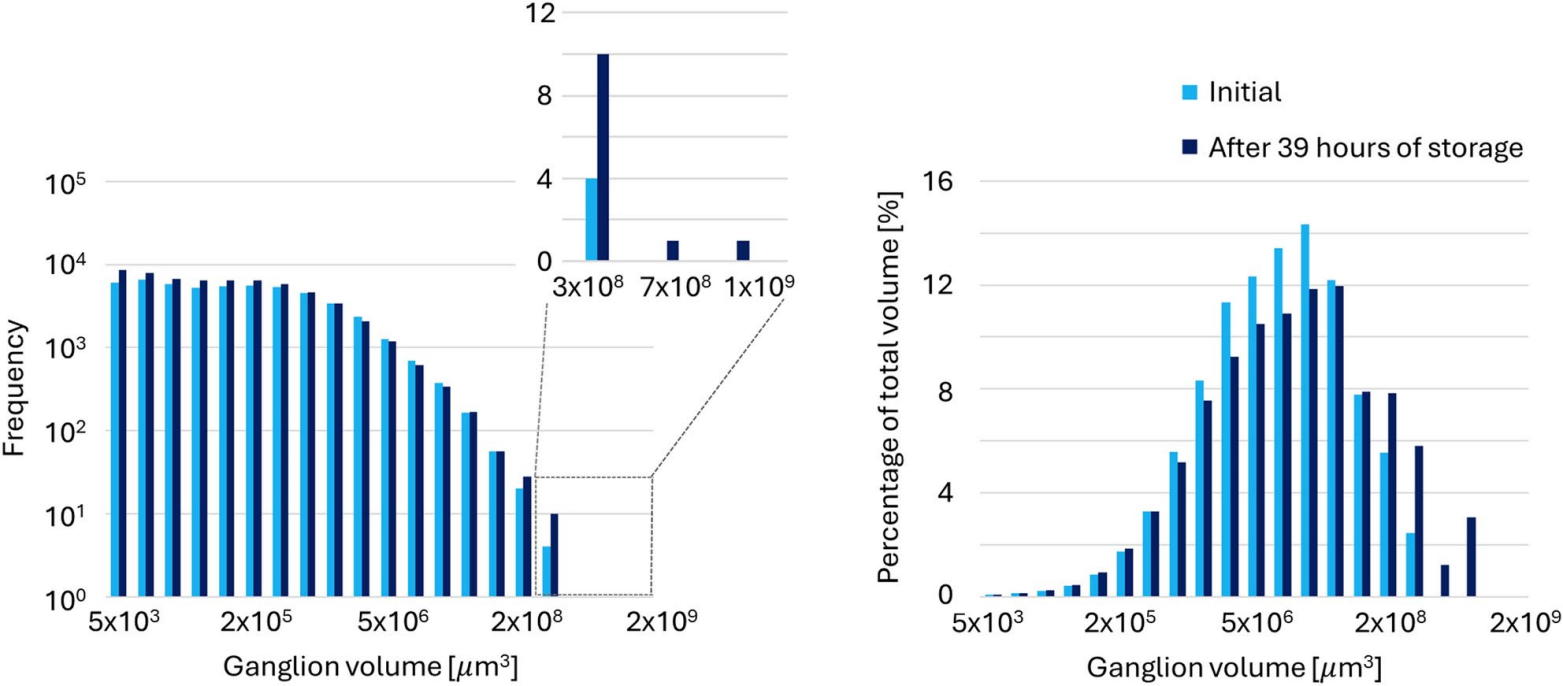
Hydrogen ganglion volume distribution for the first no-flow period: immediately after pausing the flow during drainage at the end of $f_{H2}$ = 0.1 (light blue), and after 39 h of storage (dark blue). The left plot shows the frequency distribution of ganglia sizes. The right plot displays the total volume of the ganglia in each bin relative to the total volume of all hydrogen ganglia.

**Fig. 10:**
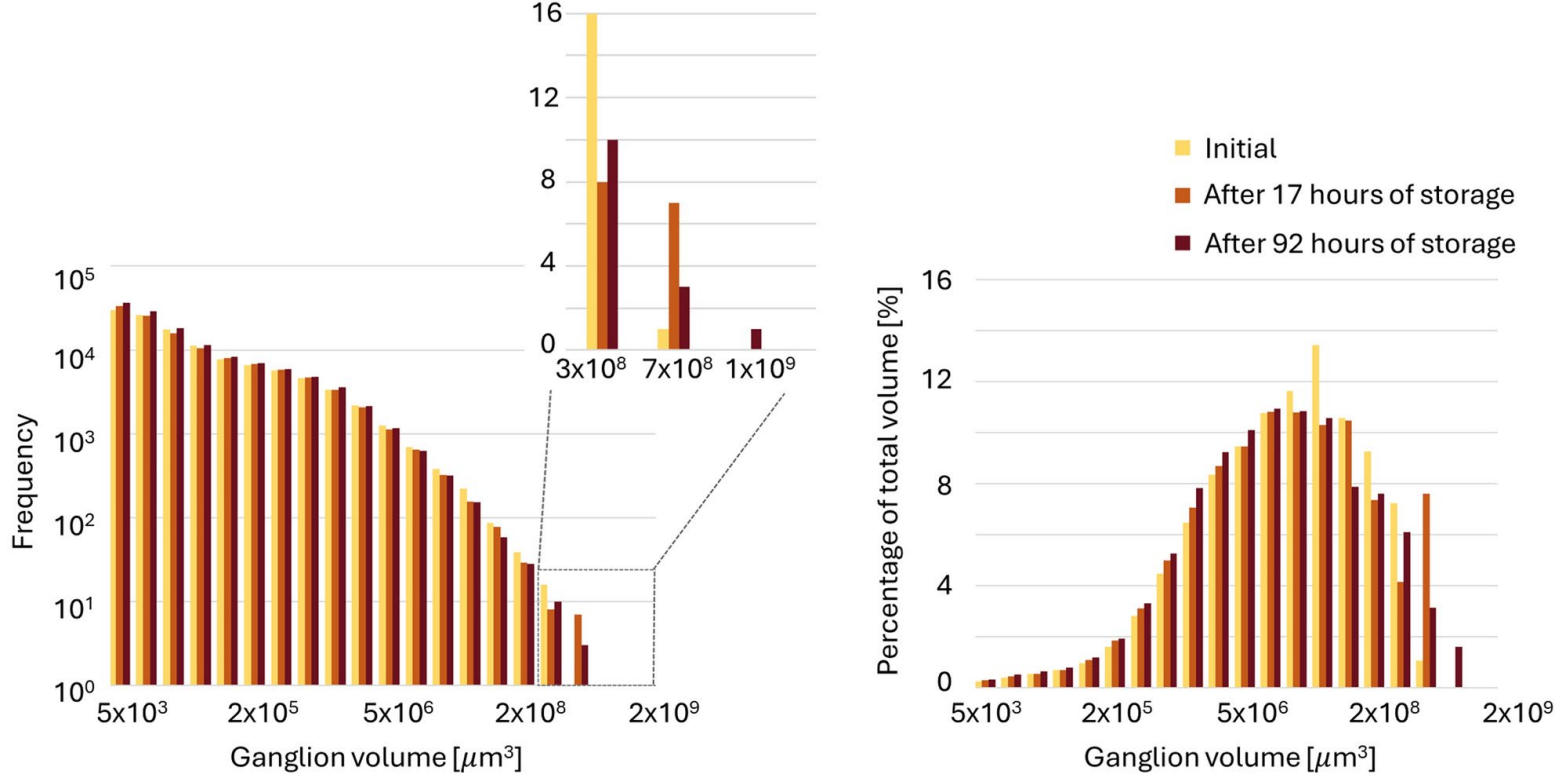
Hydrogen ganglion volume distribution during the second no-flow period: immediately after the end of imbibition relative permeability experiment at $f_{H2}$ = 0 (yellow), after 17 h of storage (light brown) and after 92 h of storage (dark brown). The left plot shows the frequency distribution of ganglia sizes. The right plot displays the total volume of the ganglia in each bin relative to the total volume of all hydrogen ganglia.

Figures 9 and 10 show the evolution of the volume distributions of disconnected hydrogen ganglia during the two no-flow periods and provide evidence that Ostwald ripening occurred. Figure 4 shows numerous disconnected ganglia at the start of the first no-flow period. This is contrary to the results by Goodarzi et al.^28^, where a single large ganglion dominated the total hydrogen volume before storage. It must be noted that the connectivity of ganglia may not be detected if the connections are smaller than the scanning resolution. The study of Goodarzi et al.^28^ used a slightly higher scanning resolution than the resolution of this study (6.5 μm). From Fig. 9, it can be observed that at the end of the first no-flow period, the quantities of both the smallest and the largest ganglia increase at the expense of the mid-sized ganglia. Two new large ganglia form, as shown in the subplot, which significantly contribute to the total volume, as indicated in the relative distribution. Additionally, the relative distribution reveals a rightward shift in the mode, indicative of Ostwald ripening^32^. The increase in the number of small ganglia and the formation of new large ganglia are likely due to mid-sized ganglia breaking apart into several smaller ganglia. This fragmentation occurs due to volume loss from dissolution and diffusion toward low $P_c$ regions, leading to the formation of larger ganglia. Small ganglia do not always completely disappear as $P_c$ may drop when the small ganglia migrate into larger pore spaces^36^, which may be allowed by this fragmentation. Furthermore, it is likely that the process of Ostwald ripening has not yet fully developed after 39 h. This hypothesis can be substantiated by estimating the time scale to reach local equilibrium using Eq. 8, which is 265 h, or 11 days. The calculation assumes a length (*l*) of 1 mm^36^, a throat radius (*r*) of 10 × 10$^{-6}$ m^42^, a gas saturation ($S_{gr}$) of 0.28, which is the hydrogen saturation when the flow is stopped, a diffusion coefficient (*D*) of 9.35 $\times$ 10$^{-10}$ m$^2$/s (which is the value for hydrogen in water at 25 °C corrected by the porosity) and Henry’s constant (*H*) is 7.8 $\times$ 10$^{-6}$ mol/m$^3$ $\cdot$Pa^43^. Please note that this time scale can be considered an overestimate because the equation estimates an equilibrium state in which all trapped gas is displaced, whereas in reality, only a fraction needs to relocate to achieve equilibrium^36^.

Figure 10 shows the changing ganglion volume distribution after storage following the end of imbibition. The subplot highlights the formation of new, larger ganglia over time. Additionally, there is a noticeable decrease in the quantity of the larger half of the mid-sized ganglia, accompanied by an increase in the number of small ganglia, possibly due to fragmentation similar to the process described for the first (drainage) no-flow period. However, it is important to note that the results of the second (post-imbibition) no-flow period are more prone to error because the images taken after 17 h and 92 h of storage were of relatively poor quality for unknown reasons. Although image processing remains possible, inconsistent CT values prevent the use of equal threshold values for the different scans. Therefore, manual segmentation was applied by initially determining the threshold values through a visual inspection, followed by iterative adjustment to ensure the resulting saturation closely matched the most recent macro-scale measurements, minimizing potential errors from the lower scan quality. However, since the pore-scale scan covers only a 1 cm section of the core (Fig. 1), potential influx or outflux over time from adjacent core sections makes this validation method less reliable.

**Fig. 11:**
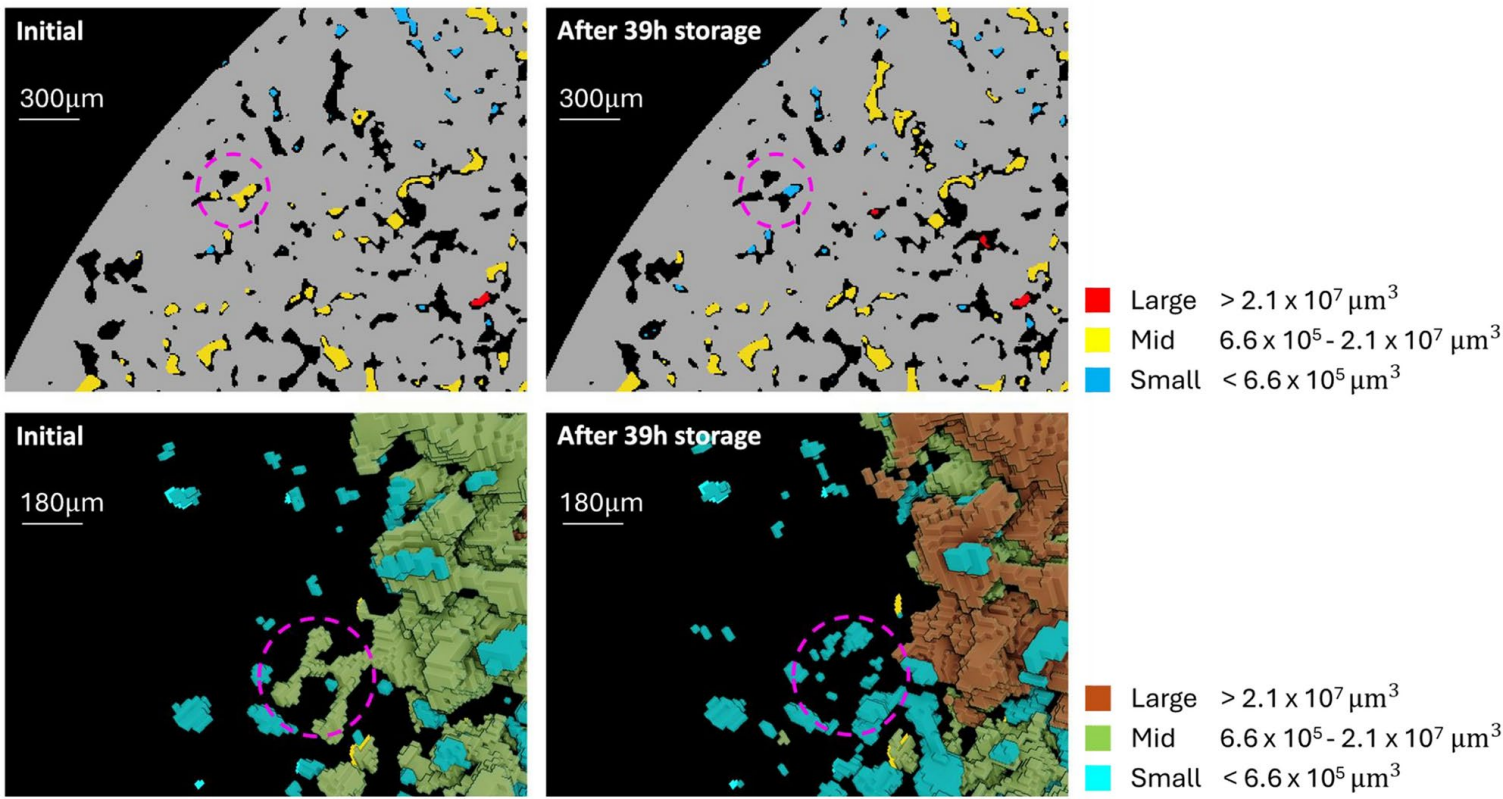
2D slice and 3D volume of hydrogen ganglia before and after storage for the first no-flow period. The 2D images illustrate the rock (grey) with a mid-sized ganglion (yellow) highlighted within the purple circle, which transforms into a smaller ganglion (blue) after storage. In the 3D figures, it is evident that the same mid-sized ganglion fragments into multiple smaller ganglia. Additionally, a large ganglion (brown) forms after storage, visible on the right side of the 3D image.

Pore-scale visualization of shrinkage and growth of ganglia after storage offers additional evidence of the process of Ostwald ripening and can be seen in Fig. 11. To visualise the concept of growth of large ganglia at the expense of fragmented mid-size ganglia, the ganglia are sieved into small, mid-size, and large ganglia and are given a different color. The ranges are based on the volume frequency distributions, where the small and large ganglia increase in frequency and the mid-size ganglia decrease in frequency.

## Conclusion

In this study, hydrogen-brine multiphase flow was visualized and characterized, using X-ray CT, in a homogeneous vertically oriented Berea sandstone at both the pore- and core-scale. A 17 cm long core with a diameter of 10.5 mm was used and the experiments were conducted at 25$^{\circ }$C and 50 bar. Low-resolution images (175 μm) were taken of the entire core, while high-resolution images (6.5 μm) were taken of a 1 cm long section in the middle of the core. Steady-state relative permeability hysteresis was measured for a single drainage and imbibition cycle under capillary-dominated conditions. Two no-flow periods were incorporated in the experiment to characterize hydrogen redistribution during periods of hydrogen storage. The main findings are listed below:

- Low hydrogen relative permeability was measured with an end-point relative permeability of 0.043 at $S_w=0.56$, and a residual gas saturation of 0.32 was found. These values are consistent with values reported in previous studies and help reduce the considerable uncertainty currently associated with UHS, as relative permeability is a crucial input parameter for reservoir simulations.
- Despite extensive pre-equilibration before the start of the experiment, significant dissolution of hydrogen in brine occurred during both drainage and imbibition due to elevated pressures, and the corresponding increase in hydrogen solubility, near the inlet of the core. During drainage many disconnected hydrogen ganglia were observed further down the core which could be explained by exsolution of the dissolved hydrogen. Furthermore, during imbibiton, dissolution led to the formation of preferential flow paths near the inlet of the core and eventually removed most of the trapped hydrogen in the final stage of the experiment. These observations highlight the complex effects of hydrogen dissolution in brine and emphasize the importance of accounting for dissolution when simulating UHS, especially in heterogeneous systems where local pressure variations can lead to complex flow and transport behavior.
- Based on mass balance, it was estimated that, next to the direct removal of hydrogen by dissolution, an additional 0.14 PV of hydrogen was removed from the core after an extended period of no-flow. This could possibly be (partly) attributed to the re-connection of disconnected ganglia by the dissolution-driven process of Ostwald ripening. Further evidence for the process of Ostwald ripening is provided by the ganglion volume distributions and 3D pore-scale visualization, which showed fragmentation of mid-size hydrogen ganglia and the growth of a few larger ganglia. To further improve modeling capabilities for UHS, the effect of Ostwald ripening needs to be considered, as it can reduce trapped hydrogen saturations and relative permeability hysteresis, which will be favorable for UHS.
- Future research could compare relative permeability core flooding experiments with and without storage time between drainage and imbibition cycles to experimentally study the effect of Ostwald ripening on relative permeability hysteresis. Additionally, investigating longer storage times with intermediate imaging could provide insights into the progression toward different equilibrium states. Furthermore, increasing the resolution of CT images would enable interfacial curvature analysis, helping to reveal the correlation between local capillary pressure and Ostwald ripening. Since the current experimental apparatus did not allow for high-temperature experiments, future experiments should, next to high-pressure, be conducted under high-temperature conditions to more accurately assess the impact of dissolution-driven processes during UHS.

## Supplementary Information

Supplementary Information.

## Data Availability

The X-ray CT images and experimental pressure data are available to be shared upon request. Please contact the corresponding author (maartje.boon@mib.uni-stuttgart.de).
