# Supplementary material for: Multiscale experimental study of H[image]/brine multiphase flow in porous rock characterizing relative permeability hysteresis, hydrogen dissolution, and Ostwald ripening
Source: Sci Rep. 2024 Dec 4;14:30170. doi: 10.1038/s41598-024-81720-4 (PMC11615215; doi:10.1038/s41598-024-81720-4)
Supplement: Supplementary file 1 — Supplementary Information. [file 41598_2024_81720_MOESM1_ESM.pdf]

Supporting Information: Multiscale experimental study of H_2/brine multiphase flow in porous rock characterizing relative permeability hysteresis, hydrogen dissolution, and Ostwald ripening

Maartje Boon[1,*], Tim Rademaker[2], Chandra Widyananda Winardhi[3], and Hadi Hajibeygi[2]

[1]University of Stuttgart, Institute of Applied Mechanics, Stuttgart, 70569, Germany
[2]Delft University of Technology, Faculty of Civil Engineering and Geosciences, Delft, 2600 GA, The Netherlands
[3]Ghent University, Department of Geology, B-9000, Ghent, Belgium
[*]maartje.boon@mib.uni-stuttgart.de

1 Pore-size distribution

The pore-size distribution of the Berea sandstone used in the experiment can be seen in Supplementary figure 1. It was obtained from high resolution CT images (6.5 μm voxel size) of a 1 cm long section in the middle of the core.

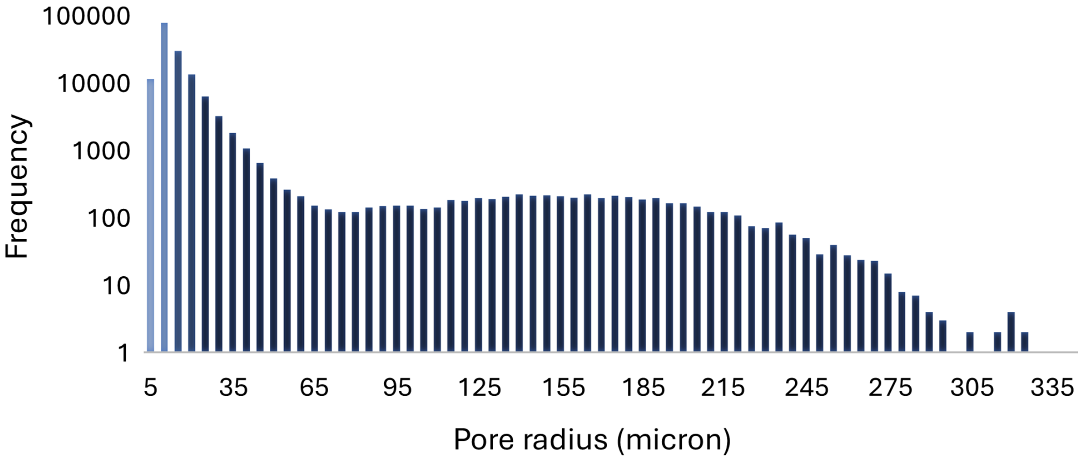

Supplementary figure 1. Pore size distribution of the Berea Sandstone rock sample obtained using high-resolution CT imaging of a 1 cm long section at the center of the core. The light blue shade indicates a larger possible error. These pore sizes are close to the voxel size (6.5 μm) and could, therefore, potentially be attributed to noise.

2 Overview of experimental studies

Study	Lithology	Core dimensions [cm] (D) / [cm] (L)	Temperature [°C]	Pressure [bar]	Porosity (%)	Permeability [mD]
Yekta et al., 2018[1]	Vosges Sandstone	1.5 / 6.1	20-45	55-100	19	44
Jha et al., 2021[2]	Gosford Sandstone	0.5 / 1.5	20	1	18	-
Al-Yaseri et al., 2022[3]	Fontainebleau Sandstone	3.9 / 5.5	25	4	10	190
Rezaei et al., 2022[4]	Sandstones & Carbonate	3.8 / 8.9-9.5	80	1-206.8	10.5-16.4	3-34
Boon et al., 2022[5]	Berea Sandstone	3.8 / 17	18	100	20	203
Lysyy et al., 2022[6]	Berea Sandstone	3.8 / 27.6	30	30	18	107
Thaysen et al., 2023[7]	Clashach Sandstone	0.5 / 5.4-5.7	20	20-70	13-14	-
Jangda et al., 2023[8]	Bentheimer Sandstone	0.6 / 2.7	50	100	23	2900
Zhang et al., 2023[9]	Bentheimer Sandstone	0.6 / 2.9	25	10	-	-
Goodarzi et al., 2024[10]	Bentheimer Sandstone	1.3 / 6.0	25	10	22.6	1400
Higgs et al., 2024[11]	Bentheimer Sandstone	0.6 / 1.3	25	21.3	23	653
This study	Berea Sandstone	1.0 / 17	25	50	18.3	104

Supplementary table 1. Rock type and experimental conditions for previous relative permeability and residual trapping studies. Empty fields indicate that the data was either not determined in the study or not provided by the authors. This table is based on data gathered by Higgs et al.[11] and has been modified and extended.

Study	Capillary number range [-]	Displacement methodology	Flow orientation	Imaging technique
Yekta et al., 2018[1]	1.17-1.25 x 10^{-8} (d)	Steady-state	Vertical	No
Jha et al., 2021[2]	2.49 x 10^{-10} - 3.68 x 10^{-8} (i)	Unsteady-state	Vertical	Micro-CT
Al-Yaseri et al., 2022[3]	3.54 x 10^{-8} (d)	Unsteady-state	Vertical	NMR
Razaei et al., 2022[4]	-	Unsteady-state	Vertical	No
Boon et al., 2022[5]	9.1 x 10^{-9} - 1.1 x 10^{-6} (d)	Steady-state	Horizontal	Medical CT
Lysyy et al., 2022[6]	1.88 x 10^{-9} - 1.74 x 10^{-7} (d)	Steady-state	Vertical	X-ray (unspecified)
Thaysen et al., 2023[7]	1.2 x 10^{-8} - 9.5 x 10^{-6} (i)	Unsteady-state	-	Micro-CT
Jangda et al., 2023[8]	4.2 x 10^{-9} - 2.3 x 10^{-6} (d)	Unsteady-state	Vertical	Micro-CT
Zhang et al., 2023[9]	2.55 x 10^{-9} - 2.62 x 10^{-7}	Unsteady-state	Vertical	Micro-CT
Goodarzi et al., 2024[10]	1.93 x 10^{-7} - 5.79 x 10^{-9} (d)	Unsteady-state	Vertical	Micro-CT
Higgs et al., 2024[11]	2.92 x 10^{-7} - 2.92 x 10^{-5} (d)	Steady-state	Vertical	Micro-CT
This study	1.3 x 10^{-8} - 1.3 x 10^{-6} (d)	Steady-state	Vertical	Micro-CT

(d) Determined with Darcy velocity (i) Determined with Interstitial velocity

Supplementary table 2. Experimental methods of previous relative permeability and residual trapping studies. Empty fields indicate that the data was either not determined in the study or not provided by the authors. This table is based on data gathered by Higgs et al.[11] and has been modified and extended.

Study	Initial gas saturation [-]	Residual gas saturation [-]	Hydrogen end-point relative permeability [-]	Max. pressure drop [bar]
Yekta et al., 2018[1]	0.59-0.6	-	0.035-0.044	0.9 (D) - 2.0 (D)
Jha et al., 2021[2]	0.65 (x)	0.41 (x)	-	-
Al-Yaseri et al., 2022[3]	0.04 (x)	0.02 (x)	-	-
Rezaei et al., 2022[4]	0.22-0.28	-	0.260-0.360	-
Boon et al., 2022[5]	0.36 (y)	0.24-0.17 (y)	0.019	0.6 (D) - 4.9 (I)
Lysyy et al., 2022[6]	0.41 (x)	0.36 (x)	0.037-0.083	0.3 (D) - 4.2 (I)
Thaysen et al., 2023[7]	0.50-0.53	0.1-0.21	-	-
Jangda et al., 2023[8]	0.36 (x) - 0.36 (y)	0.20 (x) - 0.25 (y)	-	-
Zhang et al., 2023[9]	0.53 (x)	0.07 (x)	-	-
Goodarzi et al., 2024[10]	0.30 (x)	0.10 (x)	-	-
Higgs et al., 2024[11]	0.76 (x)	0.40 (x)	0.049	0.1 (D) - 0.9 (I)
This study	0.44 (y)	0.32 (y)	0.043	18.0 (D) - 15.9 (I)

(x) Determined with non-equilibrated aeqeous phase (y) Determined with equilibrated aeqeous phase (D) Drainage (I) Imbibition

Supplementary table 3. Results from previous relative permeability and residual trapping studies. Empty fields indicate that the data was either not determined in the study or not provided by the authors. This table is based on data gathered by Higgs et al.[11] and has been modified and extended.

3 Pressure profile - last step of the imbibition experiment

After the second no-flow period, 30 PV of equilibrated brine were injected into the core. The pressure evolution at the inlet and outlet can be seen in Supplementary figure 2.

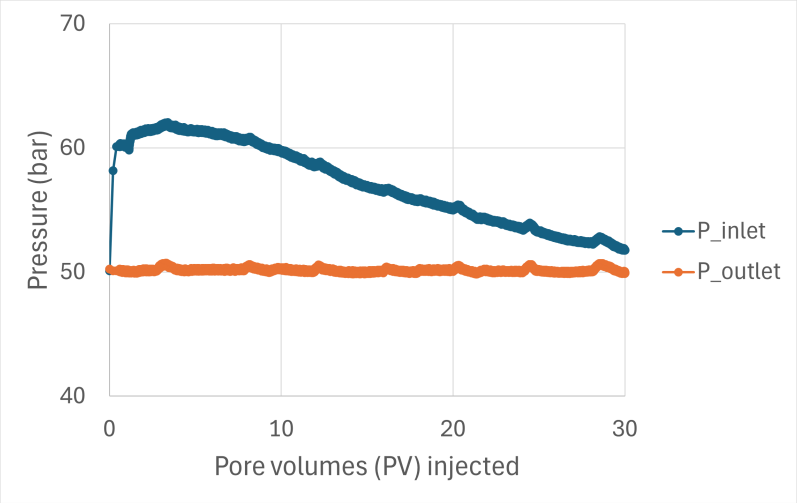

Supplementary figure 2. Pressure versus pore volume graph for the last step of the imbibition experiment. 30 pore volumes (PV) of pre-equilibrated brine were injected after a no-flow period.

References

1. Yekta, A. E., Manceau, J.-C., Gaboreau, S., Pichavant, M. & Audigane, P. Determination of hydrogen–water relative permeability and capillary pressure in sandstone: Application to underground hydrogen injection in sedimentary formations. *Transp. Porous Media* **122**, 333–356, DOI: 10.1007/s11242-018-1004-7 (2018).
2. Jha, N. K. *et al.* Pore-scale investigation of hydrogen injection in sandstone via x-ray micro-tomography. *Int. J. Hydrog. Energy* **46**, 34822–34829, DOI: 10.1016/j.ijhydene.2021.08.042 (2021).
3. Al-Yaseri, A. *et al.* Initial and residual trapping of hydrogen and nitrogen in fontainebleau sandstone using nuclear magnetic resonance core flooding. *Int. J. Hydrog. Energy* DOI: 10.1016/j.ijhydene.2022.05.059 (2022).
4. Rezaei, A. *et al.* Relative permeability of hydrogen and aqueous brines in sandstones and carbonates at reservoir conditions. *Geophys. Res. Lett.* **49**, e2022GL099433, DOI: 10.1029/2022GL099433 (2022).
5. Boon, M. & Hajibeygi, H. Experimental characterization of H_2/water multiphase flow in heterogeneous sandstone rock at the core scale relevant for underground hydrogen storage (uhs). *Sci. Reports* **12**, 14604, DOI: 10.1038/s41598-022-18759-8 (2022).
6. Lysyy, M., Føyen, T., Johannesen, E. B., Fernø, M. & Ersland, G. Hydrogen relative permeability hysteresis in underground storage. *Geophys. Res. Lett.* **49**, e2022GL100364, DOI: 10.1029/2022GL100364 (2022).
7. Thaysen, E. M. *et al.* Pore-scale imaging of hydrogen displacement and trapping in porous media. *Int. J. Hydrog. Energy* **48**, 3091–3106, DOI: 10.1016/j.ijhydene.2022.10.153 (2023).
8. Jangda, Z. *et al.* Pore-scale visualization of hydrogen storage in a sandstone at subsurface pressure and temperature conditions: Trapping, dissolution and wettability. *J. Colloid Interface Sci.* **629**, 316–325, DOI: 10.1016/j.jcis.2022.09.082 (2023).
9. Zhang, Y. *et al.* Pore-scale observations of hydrogen trapping and migration in porous rock: Demonstrating the effect of ostwald ripening. *Geophys. Res. Lett.* **50**, e2022GL102383, DOI: 10.1029/2022GL102383 (2023).
10. Goodarzi, S., Zhang, Y., Foroughi, S., Bijeljic, B. & Blunt, M. J. Trapping, hysteresis and ostwald ripening in hydrogen storage: A pore-scale imaging study. *Int. J. Hydrog. Energy* **56**, 1139–1151, DOI: 10.1016/j.ijhydene.2023.12.029 (2024).
11. Higgs, S. *et al.* Direct measurement of hydrogen relative permeability hysteresis for underground hydrogen storage. *Int. J. Hydrog. Energy* **50**, 524–541, DOI: 10.1016/j.ijhydene.2023.07.270 (2024).
